# Antiplatelet Therapy in Chronic Coronary Artery Disease Patients With a History of Angioplasty. When is Aspirin Not Enough? A Systematic Review

**DOI:** 10.31083/RCM44227

**Published:** 2025-09-24

**Authors:** Stylianos Fiflis, Michail Papamichalis, Andrew Xanthopoulos

**Affiliations:** ^1^Department of Cardiology, University Hospital of Larissa, Faculty of Medicine, University of Thessaly, 41110 Larissa, Greece

**Keywords:** chronic coronary syndrome, history of percutaneous coronary intervention, aspirin, dual antiplatelet therapy, ticagrelor monotherapy, clopidogrel monotherapy

## Abstract

**Background:**

Antiplatelet therapy represents a cornerstone of secondary prevention in patients with chronic coronary syndrome (CCS) who have undergone percutaneous coronary intervention (PCI). However, the optimal antiplatelet regimen and optimal duration remain under investigation, as treatment must be individualized to balance the thrombotic and bleeding risks. Thus, this systematic review aimed to present the most recent evidence on antiplatelet strategies in chronic coronary syndrome patients with prior PCI, highlighting findings relevant to subgroups with increased thrombotic risk.

**Methods:**

A systematic search of the PubMed database, the Cochrane Library, and ClinicalTrials.gov was conducted up to 29 May 2025. Studies were screened and selected based on predefined eligibility criteria. A total of 14 studies were included and were synthesized narratively.

**Results:**

Extended dual antiplatelet therapy (DAPT) with ticagrelor plus aspirin, compared to aspirin alone, improved primary outcomes in 5101 patients with stable coronary disease and diabetes mellitus (hazard ratio (HR) 0.81; 95% confidence interval (CI), 0.71–0.93; *p* = 0.003), and reduced major adverse cardiovascular events (HR 0.85; 95% CI, 0.75–0.96; *p* = 0.009) among 11,260 patients with history of prior myocardial infarction and additional risk factors such as multivessel coronary artery disease or chronic kidney disease. In 2431 patients, long-term clopidogrel monotherapy, compared to aspirin monotherapy, was associated with improved primary outcomes (HR 0.74; 95% CI 0.63–0.86; *p* < 0.001) along with a reduction in major bleeding (HR 0.65; 95% CI 0.47–0.90; *p* = 0.008). Long-term ticagrelor monotherapy, compared to aspirin, was associated with fewer ischemic events, as defined by the primary endpoint (HR 0.73; 95% CI 0.57–0.94; *p* = 0.014), but an increased risk of Bleeding Academic Research Consortium (BARC) type 2,3, or 5 bleeding (HR 1.52; 95% CI 1.11–2.08; *p* = 0.009). Subgroup analyses suggested benefits of extended DAPT versus aspirin in patients with peripheral artery disease (n = 246; HR 0.54; 95% CI 0.31–0.95; *p* = 0.03), in those with two or more implanted stents (n = 505; *p* = 0.02), and in patients treated for in-stent restenosis (n = 224; *p* = 0.034).

**Conclusion:**

Extended DAPT demonstrated benefits over 30 months, while clopidogrel monotherapy has shown sustained effectiveness for up to 5.8 years in CCS patients with a history of PCI. Individualized treatment based on thrombotic and bleeding risk remains essential. Large-scale randomized trials are warranted to define the populations most likely to benefit from long-term intensified antiplatelet therapy.

**The PROSPERO Registration:**

CRD420251069004, https://www.crd.york.ac.uk/PROSPERO/view/CRD420251069004.

## 1. Introduction

Antiplatelet therapy is a cornerstone of secondary prevention in patients who have undergone percutaneous coronary intervention (PCI). The standard regimen in these patients involves 6 months of dual antiplatelet therapy (DAPT) with aspirin and a P2Y12 inhibitor for chronic coronary syndrome (CCS) or 12 months for acute coronary syndrome (ACS), followed by aspirin monotherapy [1]. According to current European Society of Cardiology (ESC) guidelines, patients with CCS undergoing PCI who are at increased ischemic risk but low bleeding risk may benefit from prolonged potent antiplatelet therapy rather than standard aspirin monotherapy [2]. Alternative strategies include long-term monotherapy with clopidogrel or ticagrelor. Although clopidogrel demonstrated favorable outcomes in the CAPRIE trial, its effectiveness is limited by variable platelet reactivity in a notable proportion of patients, a phenomenon less commonly observed with more potent thienopyridines such as prasugrel and ticagrelor [3, 4]. However, use of ticagrelor has been associated with dyspnea in up to 1 in 15 patients, a side effect that may be mitigated by the use of the 60 mg dose [5]. Another option is prolonged DAPT, which offers enhanced ischemic protection through synergistic inhibition of multiple platelet activation pathways. However, this approach clearly carries increased risk of bleeding [6]. It is evident that the optimal antiplatelet therapy should be individualized to maintain protection against ischemic events without unnecessarily increasing bleeding risk, thus achieving maximal therapeutic benefit. Despite this, most clinical trials evaluate antiplatelet regimens across broad populations rather than in subgroups defined by comorbidities or high-risk clinical and anatomical features [7]. This systematic review explores the latest evidence on managing CCS, highlighting patient subgroups with a high thrombotic burden who may confer greater benefit from intensified antiplatelet regimens, such as prolonged DAPT or P2Y12 inhibitor monotherapy, compared to standard long-term aspirin monotherapy.

## 2. Methodology

### 2.1 Aim of the Review

The optimal antiplatelet strategy for patients with CCS and a history of PCI remains an area of ongoing research. According to recent ESC guidelines, aspirin is the agent of choice for secondary prevention following an initial period of DAPT. The duration of this initial DAPT may be shortened in patients at high bleeding risk or extended in those with high thrombotic burden and no significant bleeding risk factors [2]. This systematic review aims to present the current evidence on long-term antiplatelet strategies in patients with CCS and prior PCI. Specifically, it explores the use of prolonged therapy either as monotherapy with a non-aspirin antiplatelet agent or as combination antiplatelet therapy, compared with aspirin monotherapy. Although the included studies were not limited to patients at high thrombotic risk, as defined in subsection 2.6, particular emphasis was placed on findings relevant to such subgroups, where reported.

### 2.2 PICO Framework and Study Inclusion Criteria

The PICO framework was used to formulate the clinical question and guide the literature search strategy.

Population (P): Patients with CCS and a history of PCI.

Intervention (I): Long-term therapy with aspirin.

Comparison (C): Monotherapy with alternative antiplatelet agents such as clopidogrel, ticagrelor; prolonged DAPT with aspirin plus clopidogrel or plus a potent P2Y12 inhibitor.

Outcome (O): Effectiveness of more potent antiplatelet regimens in preventing ischemic events, including cardiovascular mortality, all-cause mortality, myocardial infarction, stent thrombosis, bleeding.

To be eligible for inclusion in this systematic review, studies were required to meet the following criteria: publication in English language, randomized controlled trial (RCT) design or subgroup analyses of RCT, provided that the subgroup met the inclusion criteria even if the primary trial did not. Eligible studies had to clearly document the antiplatelet regimens administered, including the duration of therapy and directly compare long-term aspirin therapy either with monotherapy using an alternative antiplatelet regimen or with DAPT in patients who had undergone PCI.

Observational studies were excluded from this review, as the aim was to include only high-quality data from randomized trials to minimize the risk of bias. Patients receiving anticoagulation were also excluded.

### 2.3 Search Strategy

**Fig. 1.**
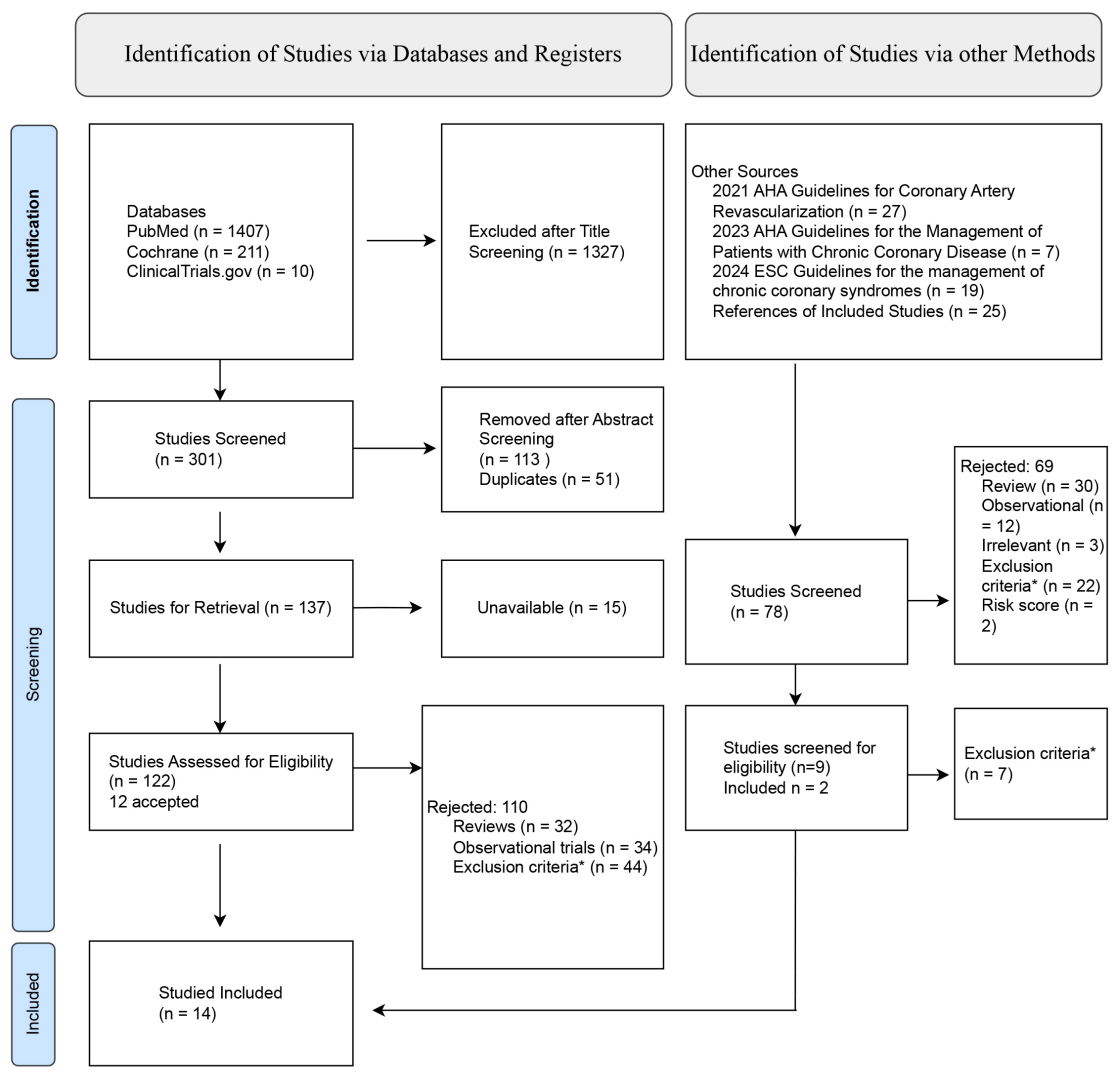
**PRISMA flowchart**. PRISMA, Preferred Reporting Items for Systematic Reviews and Meta-Analyses; AHA, American Heart Association; ESC, European Society of Cardiology. *Does not include patients with history of angioplasty, anticoagulants in the treatment strategy, does not compare experimental treatment to aspirin.

A comprehensive literature search was conducted independently by two reviewers using PubMed and the Cochrane Library. The search employed combinations of the following search terms: “PCI”, “percutaneous coronary intervention”, “angioplasty”, “prolonged DAPT”, “extended DAPT”, “intensified DAPT”, “monotherapy”, “clopidogrel”, “ticagrelor”, “prasugrel”, “P2Y12 inhibitor”, “P2Y inhibitor” and “long term”. No restrictions were applied regarding publication date or study design, but the search was limited to articles published in the English language. The final search was performed on 29 May 2025 and identified 1628 articles. After removing duplicates and screening for relevance based on predefined inclusion criteria, 14 studies were included in this review. Due to lack of institutional access, Embase was not searched. To address this limitation, additional efforts were undertaken, including manual screening of reference lists from relevant ESC and ACC guidelines on chronic coronary syndrome and coronary revascularization, a targeted search of ClinicalTrials.gov and screening the reference lists of included trials. These supplementary searches yielded 78 articles, of which 2 met the eligibility criteria [2, 8, 9]. The PRISMA 2020 flow diagram summarizing the study selection process is provided in Fig. 1.

In total, this systematic review included 11 original RCTs, 2 prespecified subgroup analyses and 1 post hoc subgroup analysis. These subgroup analyses were derived from RCTs that did not meet the predefined eligibility criteria in their primary analysis.

### 2.4 Data Extraction

From the 14 studies that were included, the following data were independently extracted by two reviewers: country of origin, study design, total number of enrolled patients, baseline characteristics and comorbidities such as age, sex, diabetes mellitus, smoking status and prior myocardial infarction (MI). Additional details included the indication for PCI, type of stent used, target vessel for revascularization, timing of randomization to treatment arms, and details of the intervention and comparator regimens, including duration of therapy. Study endpoints such as all-cause mortality, cardiovascular mortality, MI, stent thrombosis and bleeding events were recorded, along with follow-up duration and study limitations. The extracted data were organized into structured tables to enable systematic comparison of study characteristics and clinical outcomes. Disagreements between reviewers were resolved by consensus.

### 2.5 Quality Assessment

Two reviewers independently assessed the risk of bias for each study using the Cochrane Risk of Bias 2 (RoB 2.0) tool. Discrepancies were resolved through discussion. Risk of bias was evaluated across five domains, as low, some concerns, or high. Of the 14 included studies, 10 were judged to be at low risk, 3 studies had some concerns, and 1 was judged to be at high risk. Full domain-level assessments for each study are provided in the **Supplementary Fig. 1**.

### 2.6 Data Synthesis

Given the substantial heterogeneity in study designs, patient populations, clinical endpoints, and follow-up durations, quantitative meta-analysis was not feasible. Instead, a narrative synthesis was conducted, focusing on study design, population characteristics, primary outcomes, and subgroup analyses related to alternative long-term antiplatelet therapies after PCI, compared to aspirin monotherapy. The results were further organized according to the antiplatelet regimens administered.

### 2.7 Definitions

Standard DAPT was defined according to the 2023 ESC guidelines for acute coronary syndrome and the 2024 ESC guidelines for chronic coronary syndrome. Specifically, standard DAPT duration was considered to be 6 months following PCI for CCS and 12 months for ACS.

Long-term antiplatelet therapy was defined as any antiplatelet regimen administered after the completion of standard DAPT in patients with a history of PCI.

Extended DAPT referred to the continuation of DAPT beyond the standard duration.

Intensified antithrombotic therapy was defined as either switching from aspirin to a P2Y12 inhibitor or extending DAPT beyond standard duration with the intent to enhance protection against thrombotic events.

Control group was defined as the cohort receiving aspirin monotherapy.

Intervention group was defined as the cohort receiving the investigational therapy, such as long-term DAPT or P2Y12 inhibitor monotherapy compared with aspirin monotherapy.

Complex coronary artery disease was defined as PCI involving any of the following: at least three lesions treated or three stents implanted, bifurcation lesion treated with two stents, total stent length >60 mm, chronic total occlusion, or stenting of the last patent vessel.

High thrombotic risk was defined based on the 2024 ESC guidelines for the management of chronic coronary syndromes, which consider factors such as complex PCI and clinical characteristics such as diabetes mellitus, multivessel coronary artery disease (CAD), recurrent MI, polyvascular disease (CAD plus peripheral artery disease (PAD)), premature (<45 years) or accelerated (new lesion within 2-year time frame) CAD, concomitant systemic inflammatory and/or prothrombotic disease (e.g., HIV, chronic arthritis, antiphospholipoid syndrome).

## 3. Results and Discussion

### 3.1 Study Selection and Characteristics

This systematic review included 14 studies comprising a total of 77,875 CCS patients who underwent PCI. The characteristics of the enrolled patients and their comorbidities are summarized in Table 1 (Ref. [10, 11, 12, 13, 14, 15, 16, 17, 18, 19, 20, 21, 22, 23]).

**Table 1.** **Demographic and clinical characteristics of the patients**.

Trial	Number of patients	Male sex	Age	Diabetes status	Prior MI	Smoking history
Studies on extended DAPT						
ARCTIC – Interruption [10]	635/624	80%/81%	64 (IQR 57–73)/64 (IQR 57–73)	31%/36%	31%/30%	A 23%/24%
ITALIC [11]	924/926	79%/81%	(61.5 ± 11.2)/(61.6 ± 10.9)	37%/36%	14%/15%	52%/51%
OPTIDUAL [12]	695/690	81%/79%	(64.1 ± 10.8)/(64.2 ± 11.5)	30%/32%	17%/17%	A or R 61%/57%
DAPT (BMS) [13]	842/845	74%/78%	(58.9 ± 10.5)/(59.2 ± 11.1)	21%/20%	19%/21%	A or R 43%/43%
DAPT (DES) [14]	5020/4941	75%/74%	(61.8 ± 10.2)/(61.6 ± 10.1)	31%/30%	22%/21%	A or R 24%/24%
DES LATE [15]	2531/2514	69%/69%	(62.5 ± 10)/(62.3 ± 10.1)	28%/28%	4%/3%	A 27%/28%
NIPPON [16]	1653/1654	79%/78%	(67.2 ± 9.9)/(67.4 ± 9.6)	38%/37%	11%/12%	A or R 60%/58%
REAL-LATE and ZEST-LATE [17]	1537/1344	70%/69%	(62 ± 9.8)/(61.9 ± 9.9)	25%/27%	3%/3%	A 29%/32%
PRODIGY [18]	987/983	77%/76%	(67.8 ± 11)/(67.9 ± 11)	24%/23%	27%/26%	A 22%/25%
PEGASUS-TIMI 54 [19]	ASA+T 90 mg 5612/ASA+T 60 mg 5658/ASA 5621	DES 79.9%, BMS 77.5%	65 (IQR 58–71)	DES 32%, BMS 29%	DES 16.7%, BMS 14.3%	A DES 17%, BMS 17%
THEMIS-PCI [20]	5101/5194	69%/69.3%	66 (IQR 61–72)	100%	0%	A 12%/11%
Studies on long-term P2Y12 inhibitor monotherapy						
HOST-EXAM Extended [21]	2431/2286	74%/75%	(63.3 ± 10.8)/(63.3 ± 10.7)	33%/33%	16%/15%	A 19.7%/21%
SMART-CHOICE 3 [22]	2752/2754	82%/18%	66 (IQR 58–73)/65 (IQR 58–73)	40%/41%	46.6%/46.1%	A 16%/18%
GLOBAL LEADERS [23]	5308/5813	77%/77%	(63.7 ± 10.2)/(64.1 ± 10)	24%/24%	21%/22%	A 26%/26%

Data are presented as (intervention/control group). A, active; R, recent; MI, myocardial infarction; ASA, aspirin; T, ticagrelor; IQR, interquartile range; DES, drug eluting stent; BMS, bare metal stent; DAPT, dual antiplatelet therapy; TIMI, thrombolysis in myocardial infarction.

Long-term aspirin monotherapy was compared with alternative therapeutic strategies, including DAPT with a P2Y12 inhibitor in 11 studies and P2Y12 inhibitor monotherapy in 3 studies. Table 2 (Ref. [10, 11, 12, 13, 14, 15, 16, 17, 18, 19, 20, 21, 22, 23]) summarizes the key design features of the included studies and the antiplatelet regimens administered during both the initial and long-term periods.

**Table 2.** **Study design and antiplatelet strategies**.

Trial	Country	Trial type	Randomization	Follow up	Initial regimen	Long term regimen
Studies on extended DAPT						
ARCTIC – Interruption [10]	France	Open label, superiority RCT	12 m after initial DAPT	18 m	12 m DAPT	18 m DAPT/ASA
ITALIC [11]	France	Open label, noninferiority RCT	6 m after initial DAPT	24 m	6 m DAPT	24 m DAPT/ASA
OPTIDUAL [12]	France	Open label, superiority RCT	12 ± 3 m after initial DAPT	36 m	12 m DAPT	48 m DAPT/ASA
DAPT (BMS) [13]	International	Double blind, superiority RCT	12 m after initial DAPT	33 m after randomization	12 m DAPT	30 m DAPT 112 pts with prasugrel, 730 pts with clopidogrel/ASA
DAPT (DES) [14]	International	Double blind, superiority RCT	12 m after initial DAPT	33 m after randomization	12 m DAPT	30 m DAPT 1745 pts with prasugrel, 3275 with clopidogrel/ASA
DES LATE [15]	Korea	Open label, superiority RCT	12 m after initial DAPT	24.7–50.7 m after randomization	12 m DAPT	36 m DAPT/ASA
NIPPON [16]	Japan	Open label, noninferiority RCT	Shortly after PCI	361–540 days	6 m DAPT	18 m DAPT/ASA
REAL-LATE and ZEST-LATE [17]	Korea	Open label, superiority RCT	12.8 m after PCI	28–37 m after PCI	12 m DAPT	24 m DAPT/ASA
PRODIGY [18]	Italy	Open label, superiority RCT	30 ± 5 days after PCI	2 years after PCI	6 m DAPT	24 m DAPT/ASA
PEGASUS-TIMI 54 [19]	International	Prespecified analysis of Double blind superiority RCT	1–3 years after MI	33 m	Initial regimen NS	ASA and 1:1:1 ticagrelor 90 mg, ticagrelor 60 mg, placebo
THEMIS-PCI [20]	International	Prespecified analysis of Double blind superiority RCT	1–12 m after PCI	3.3 years	Initial regimen NS	After randomization aspirin with or without ticagrelor
Studies on long-term P2Y12 inhibitor monotherapy						
HOST-EXAM Extended [21]	Korea	Open label, superiority RCT	12 ± 6 m after initial DAPT	5.8 years	12 ± 6 m DAPT	clopidogrel/ASA
SMART-CHOICE 3 [22]	Korea	Open label, superiority RCT	17.5 m after PCI	2.3 years	DAPT with	clopidogrel/ASA
					Clopidogrel 3431 pts	
					Prasugrel 645 pts	
					Ticagrelor 1430 pts	
GLOBAL LEADERS [23]	International	Post hoc analysis of Open label superiority RCT	Shortly after PCI	2 years	1 m DAPT followed by 11 m ticagrelor/12 m DAPT with clopidogrel or ticagrelor	After 12 m: ticagrelor/ASA

Data are presented as (intervention/control group). ASA, aspirin; NS, not stated; m, months; pts, patients; RCT, randomized controlled trial; DAPT, dual antiplatelet therapy; MI, myocardial infarction; PCI, percutaneous coronary intervention; DES, drug eluting stent; BMS, bare metal stent; TIMI, thrombolysis in myocardial infarction.

The angioplasty details, including the indication for the procedure, target vessel, and type of stent implanted are demonstrated in Table 3 (Ref. [10, 11, 12, 13, 14, 15, 16, 17, 18, 19, 20, 21, 22, 23]).

**Table 3.** **Details of angioplasty procedure**.

Trial	PCI indication	Target vessel	Stent type
Studies on extended DAPT			
ARCTIC – Interruption [10]	Elective	LCA 18/23	1st gen 43%/40%
		LAD 342/325	2nd gen 62%/64%
		Cx 209/181	
		RCA 191/222	
ITALIC [11]	SA 41%	LAD 658/669	2nd gen Everolimus
	SI 15%	Cx 436/456	
	UA 20%	RCA 474/489	
	NSTEMI 16%		
	STEMI 7%		
OPTIDUAL [12]	SA 240/207	LAD 397/443	Sirolimus 214/186
	SI 138/151	Cx 225/214	Paclitaxel 164/169
	ACS 239/262	RCA 280/268	Zotarolimus 89/114
	Other 78/70		Everolimus 540/522
			Other 69/69
DAPT (BMS) [13]	SA 199/198	LCA 0/1	BMS 100%
	ACS 572/574	LAD 308/306	
	Other 71/73	Cx 206/207	
		RCA 437/452	
DAPT (DES) [14]	SA 1882/1870	LCA 55/55	Everolimus 2345/2358
	ACS 2148/2103	LAD 2715/2586	Paclitaxel 1350/1316
	Other 990/968	Cx 1473/1506	Zotarolimus 642/622
		RCA 2153/2057	Sirolimus 577/541
DES LATE [15]	SA 1011/956	LCA 112/90	Sirolimus 1566/1551
	ACS 1512/1551	LAD 1781/1768	Paclitaxel 738/709
	Other 8/7	Cx 715/651	Zotarolimus 682/664
		RCA 976/972	Everolimus 427/364
			Other 190/210
NIPPON [16]	SA 734/805	LCA 16/7	Nobori DES
	ACS 552/527	LAD 998/981	
	Other 275/268	Cx 374/381	
		RCA 515/524	
REAL-LATE and ZEST-LATE [17]	SA 514/500	LCA 55/44	Sirolimus 1057/1052
	UA 543/559	LAD 912/921	Paclitaxel 456/439
	NSTEMI 145/144	Cx 372/334	Zotarolimus 350/347
	STEMI 155/141	RCA 533/546	Other 9/9
PRODIGY [18]	SA 257/250	LCA 55/56	3rd gen thin-strunt BMS 246/246
	ACS 732/733	LAD 518/518	Everolimus 248/245
	STEMI 321/327	Cx 321/318	Paclitaxel 245/245
		RCA 346/363	Zotarolimus 248/247
PEGASUS-TIMI 54 [19]	STEMI 9552	Multivessel CAD: ASA+T 90 mg 66.7%/ASA+T 60 mg 67%/ASA 67%	BMS 51%, DES 49%
	NSTEMI 6609		1st gen DES 2289
	Other 712		2nd gen DES 4539
			Unspecified DES 1466
THEMIS-PCI [20]	Stable CAD	NS	(DES 3371, BMS 1730)/(DES 3437, BMS 1757)
Studies on long-term P2Y12 inhibitor monotherapy			
HOST-EXAM Extended [21]	SA 620/593	LCA 127/112	1st gen 2%/1%
	SI 52/61	Bifurcation 261/232	2nd gen 96%/97%
	ACS 1759/1631	Two vessel disease 763/716	
		Three vessel disease 439/428	
SMART-CHOICE 3 [22]	CCS 672/662	LCA 227/198	DES
	UA 797/823	LAD 2081/1991	
	NSTEMI 678/652	Cx 1191/1155	
	STEMI 605/617	RCA 1285/1285	
GLOBAL LEADERS [23]	CCS 2742/3228	LCA 139/136	Biolimus A9 eluting stents
	UA 702/695	LAD 2670/3028	
	NSTEMI 1140/1139	Cx 1677/1824	
	STEMI 724/751	RCA 1990/2109	

Data are presented as (intervention/control group). SA, stable angina; ACS, acute coronary syndrome; UA, unstable angina; SI, silent ischemia; STEMI, ST-elevation myocardial infarction; NSTEMI, Non-ST-elevation myocardial infarction; LCA, left coronary artery; LAD, left anterior descending artery; Cx, circumflex; RCA, right coronary artery; gen, generation; DES, drug eluting stent; BMS, bare metal stent; d., disease; CAD, coronary artery disease; ASA, aspirin; T, ticagrelor; PCI, percutaneous coronary intervention; NS, not stated; DAPT, dual antiplatelet therapy; TIMI, thrombolysis in myocardial infarction.

All included trials excluded patients who experienced an ischemic or bleeding event during the standard DAPT period, with the exception of the PRODIGY trial, which included such patients. The exclusion of patients with early ischemic events introduces a risk of selection bias and limits the generalizability of trial findings to a lower-risk, event-free population. Consequently, the benefits of prolonged DAPT may be underestimated in clinical settings, where early thrombotic events often prompt intensification or extension of antiplatelet therapy.

### 3.2 Efficacy and Safety of Intensified Antiplatelet Therapy

A total of nine studies including 29,345 patients evaluated whether extending DAPT to 18–48 months confers clinical benefit compared to standard duration DAPT followed by aspirin monotherapy in patients undergoing PCI [10, 11, 12, 13, 14, 15, 16, 17, 18]. Of these, 12,845 patients received extended DAPT with aspirin and clopidogrel, while 1942 patients across three studies received aspirin plus prasugrel. Only one of the nine studies, the DAPT trial, demonstrated a reduction in ischemic events with extended therapy in patients treated with drug eluting stent (DES). In that trial, treatment was continued for 30 months resulting in a significant reduction in the composite primary endpoint (HR 0.71; 95% CI 0.59–0.85; *p* < 0.001), without an increase in severe bleeding. Subgroup analyses by sex, age, body mass index, diabetes status, and smoking history did not reveal any significant interaction or enhanced benefit from extended therapy [14]. In contrast, the benefit was less evident in the BMS cohort of DAPT trial by Kereiakes *et al*. [13], where no significant reduction in the composite primary endpoint was observed (HR 0.92; 95% CI 0.57–1.47; *p* = 0.72). A post hoc interaction analysis found no statistically significant difference in treatment effect between DES and BMS groups for either stent thrombosis (interaction *p* = 0.42) or the primary endpoint (interaction *p* = 0.32), suggesting that the observed discrepancy may reflect limited statistical power in the smaller BMS subgroup rather than a true divergence in effect [13]. Nonetheless, reductions in stent thrombosis and spontaneous MI were seen in DES recipients but not in those with BMS, raising uncertainty about the underlying mechanism; particularly given that non–stent related MI reductions would be expected to occur irrespective of stent type [24]. Moreover, within the DES cohort, patients receiving everolimus-eluting stents (EES) did not appear to benefit from prolonged therapy for the primary endpoint, in contrast to those with paclitaxel-eluting stents (PES), who showed a more pronounced response [14]. This likely reflects the higher thrombotic risk associated with first-generation stents such as PES, which may have driven a greater absolute benefit.

Three other studies that compared prolonged DAPT with aspirin monotherapy did not show a benefit of extended DAPT over long-term aspirin monotherapy in terms of ischemic events, nor a significant increase in severe bleeding, also did not identify any subgroups, based on age, sex, diabetes status and ACS at presentation, who derived particular benefit [10, 11, 15]. The NIPPON study showed no overall benefit of prolonged DAPT with clopidogrel for 18 months compared to aspirin monotherapy, nor an increase in Bleeding Academic Research Consortium (BARC) type 3 or 5 bleeding. However, a significant benefit in the primary endpoint was observed exclusively in the subgroup of 505 patients who had two or more stents placed (1.2% with prolonged DAPT vs. 3.4% with standard therapy, *p* = 0.02) [16]. In the PRODIGY trial, 24-month DAPT with clopidogrel showed no overall clinical advantage and was associated with an increased risk of thrombolysis in myocardial infarction (TIMI) major bleeding compared to aspirin monotherapy. Nonetheless, long-term DAPT with clopidogrel compared to aspirin monotherapy demonstrated a significant reduction in death and MI among 224 patients treated for in-stent restenosis (*p* = 0.034), without a corresponding increase in bleeding [25]. Additionally, in a subgroup of 246 patients with PAD extended DAPT significantly reduced all-cause mortality, MI and cerebrovascular accident (HR 0.54; 95% CI 0.31–0.95; *p* = 0.03) and definite or probable stent thrombosis (HR 0.07; 95% CI 0.00–1.21, *p* = 0.01) versus aspirin monotherapy, without an associated increase in bleeding [26]. Furthermore, there was a significant reduction in definite or probable stent thrombosis in PRODIGY patients with 30% or more stenosis of the left main and/or proximal left anterior descending artery (LAD) artery, although this was accompanied by a significant increase in BARC type 3 or 5 bleeding [27].

Two studies that followed high ischemic-risk patients on 3-year DAPT with ticagrelor versus aspirin monotherapy showed significant benefit in terms of the primary composite endpoint and reduction in MI, without an increase in intracranial or fatal bleeding, though they did show a significant increase in major bleeding according to TIMI criteria. Specifically, the THEMIS-PCI trial enrolled 5101 patients with stable CAD and type 2 diabetes, demonstrating a reduction in the primary outcome with ticagrelor plus aspirin compared to aspirin alone (HR 0.81; 95% CI, 0.71–0.93; *p* = 0.003) [20]. The PEGASUS-TIMI 54 trial enrolled stable patients with a prior MI and at least one additional risk factor, such as multivessel CAD, chronic kidney disease (creatinine clearance <60 mL/min), or a history of multiple MIs [19, 28]. In this systematic review, we included the prespecified analysis of the PEGASUS-TIMI 54 trial by Bergmark *et al*. [19], which focused on 11,260 patients with a history of PCI who received extended DAPT with ticagrelor (90 mg or 60 mg) plus aspirin. This subgroup showed a reduction in the primary endpoint compared to aspirin monotherapy (HR 0.85; 95% CI, 0.75–0.96; *p* = 0.009), with an increased risk of TIMI major bleeding (HR 2.65; 95% CI, 1.90–3.68; *p* < 0.001), but no excess in fatal or intracranial bleeding [19]. To better identify patients likely to derive net clinical benefit, Magnani *et al*. [29] conducted a post hoc analysis of PEGASUS-TIMI 54. High bleeding risk was defined by the presence of baseline anemia or a history of spontaneous bleeding requiring hospitalization. Patients without either of these characteristics, classified as having low bleeding risk, extended DAPT with ticagrelor 60 mg, resulted in significantly fewer bleeding events and was associated with a 20% relative reduction in the composite of cardiovascular death, MI, or stroke compared to aspirin monotherapy [29]. Similarly, Bonaca *et al*. [30] showed in another post hoc analysis that the number of ischemic risk factors was directly associated with the clinical benefit of extended DAPT with ticagrelor among patients without high bleeding risk, as defined by Magnani *et al*. [29]. The relative risk reduction for the primary composite endpoint was 13% in those with 0–1 risk factor, 19% with two, and 23% with three or more. These ischemic risk factors included those defined in the original PEGASUS-TIMI 54 criteria, as well as diabetes and PAD. However, the findings of these two post hoc analyses are exploratory and hypothesis-generating and should be interpreted with caution. Additionally, they evaluated the overall cohort of PEGASUS-TIMI 54 cohort, which included patients without a history of PCI.

A total of three studies evaluated the efficacy and safety of long-term P2Y12 inhibitor monotherapy compared with aspirin monotherapy in patients with CCS and history of PCI [21, 22, 23]. Extended follow-up data from the HOST-EXAM study showed that in 2431 patients clopidogrel monotherapy over a median of 5.8 years was associated with improved outcomes in the primary composite endpoint (HR 0.74; 95% CI, 0.63–0.86; *p* < 0.001) and significantly reduced major bleeding (HR 0.65; 95% CI 0.47–0.90; *p* = 0.008) compared to aspirin monotherapy. These benefits were consistent across all predefined subgroups, without any significant interaction [21]. Similarly, the SMART-CHOICE 3 trial demonstrated that clopidogrel monotherapy was superior to aspirin in patients with high thrombotic burden including those with a prior MI or diabetes or complex PCI [22]. Notably, a significant interaction was observed between clinical presentation and treatment effect (*p* for interaction = 0.04), as patients without prior MI derived greater benefit from clopidogrel, reflected by a reduced incidence of major adverse cardiovascular and cerebrovascular events over 3 years (HR 0.56, 95% CI 0.39–0.81). Both trials are still undergoing extended follow-up, and final results are awaited. Due to the known variability in clopidogrel effectiveness associated with CYP2C19 loss-of-function alleles, the authors of the SMART-CHOICE 3 trial conducted an exploratory analysis to assess whether metabolic genotype influenced clinical outcomes among patients receiving clopidogrel monotherapy. Among the 731 patients who underwent genotyping, no significant differences in outcomes were observed between normal/rapid and intermediate/poor metabolizers of clopidogrel. Interestingly, a separate substudy from the PLATO trial found that clopidogrel modestly reduced leukocyte counts, independent of genotype, inflammatory biomarkers, or baseline clinical characteristics [31]. This finding raises the possibility of an off-target anti-inflammatory effect of clopidogrel, which may contribute to its clinical benefit in reducing cardiovascular events [32].

In the GLOBAL LEADERS study, extended ticagrelor monotherapy for 12 months after initial DAPT resulted in a reduction in the primary composite outcome (HR 0.73; 95% CI 0.57–0.94; *p* = 0.014), and in MI (HR 0.57; 95% CI 0.38–0.85; *p* = 0.006) compared to aspirin. No significant interaction was noted among the analyzed subgroups and although ticagrelor significantly increased BARC type 2, 3, or 5 bleedings (HR 1.52; 95% CI 1.11–2.08; *p* = 0.009), it did not increase the more serious type 3 or 5 [23]. However, because the analysis was post-hoc and non-prespecified, the findings should be interpreted with caution, given the high risk of bias.

A detailed summary of the primary endpoints, as well as myocardial infarction and stent thrombosis outcomes for each study, is provided in Tables 4,5 (Ref. [10, 11, 12, 13, 14, 15, 16, 17, 18, 19, 20, 21, 22, 23]), while bleeding outcomes are presented in Table 6 (Ref. [10, 11, 12, 13, 14, 15, 16, 17, 18, 19, 20, 21, 22, 23]).

**Table 4.** **Primary endpoint and main conclusion**.

Trial	Primary endpoint	Primary endpoint [hazard ratio (95% CI)]	Conclusion
Studies on extended DAPT			
ARCTIC – Interruption [10]	All-cause death, MI, ST, stroke, or urgent revascularisation	24 (4%)/27 (4%) [1.17 (0.68–2.03) *p* = 0.58]	No superiority of extended DAPT beyond 12 to 18 months compared to aspirin monotherapy
ITALIC [11]	All-cause death, MI, urgent TVR, stroke, and major bleeding	34 (3.7%)/32 (3.5%) [0.939 (0.580–1.522) *p* = 0.799]	Non-inferiority of 6 m of DAPT compared to 24 m of therapy
OPTIDUAL [12]	All-cause death, MI, stroke, or major bleeding	40 (5.8%)/52 (7.5%) [0.75 (0.5–1.28) *p* = 0.17]	Extension of DAPT to 18–48 months did not demonstrate superiority over aspirin monotherapy
DAPT (BMS) [13]	All-cause death, MI, stroke	33 (4.04%)/38 (4.69%) [0.92 (0.57–1.47) *p* = 0.72]	Extension of DAPT to 30 m with clopidogrel or prasugrel did not demonstrate superiority in ischemic endpoints
DAPT (DES) [14]	All-cause death, MI, stroke	211 (4.3%)/285 (5.9%) [0.71 (0.59–0.85) *p * < 0.001]	Extension of DAPT to 30 m demonstrated superiority for the primary endpoint
DES LATE [15]	All-cause death, MI, stroke	61 (2.6%)/57 (2.4%) [0.94 (0.66–1.35) *p* = 0.75]	Extension of DAPT to 24 m did not confer benefit over aspirin monotherapy for the primary endpoint
NIPPON [16]	All-cause death, MI, stroke, major bleeding	24 (1.5)/34 (2.1%) [–0.6 (–1.5–0.3) *p* = 0.24]	Non-inferiority of 6m of DAPT compared to extended 18 months DAPT
REAL-LATE and ZEST-LATE [17]	MI, CV death	20 (1.8%)/12 (1.2%) [1.65 (0.8–3.36) *p* = 0.15]	Extension of DAPT to 24 m did not provide benefit in reducing MI or all-cause mortality
PRODIGY [18]	All-cause death, nonfatal MI, stroke	100 (10.1%)/98 (10%) [0.98 (0.74–1.29) *p* = 0.91]	Extension of DAPT to 24 m did not confer benefit for the primary endpoint
PEGASUS-TIMI 54 [19]	CV death, MI, stroke	ticagrelor 90 mg vs placebo: (7.13%)/(7.98%) [0.86 (0.75–0.99) *p* = 0.042]	Both ticagrelor doses reduced the primary endpoint, with a number needed to treat (NNT) of 118 for the 90 mg dose and 85 for the 60 mg dose
		ticagrelor 60 mg vs placebo: (6.8%)/(7.98%) [0.84 (0.73–0.97) *p* = 0.016]	
THEMIS-PCI [20]	CV death, MI, stroke	367 (7.2%)/457 (8.8%) [0.81 (0.71–0.93) *p* = 0.003]	The addition of ticagrelor resulted in a significant benefit for the primary endpoint
Studies on long-term P2Y12 inhibitor monotherapy			
HOST-EXAM Extended [21]	All-cause death, nonfatal MI, stroke, readmission attributable to ACS, and BARC type 3 or greater bleeding	311 (12.8%)/387 (16.9%) [0.74 (0.63–0.86) *p * < 0.001]	Superiority of clopidogrel over long-term aspirin monotherapy was demonstrated for the primary composite endpoint
SMART-CHOICE 3 [22]	All-cause death, MI, stroke	92 (4.4%)/128 (6.6%) [0.71 (0.54–0.93) *p* = 0.013]	In high risk of recurrent ischaemic events patients, clopidogrel monotherapy results in lower risk of primary end point without an increase in bleeding
GLOBAL LEADERS [23]	All-cause death, MI, stroke	101 (1.9%)/151 (2.6%) [0.73 (0.57–0.94) *p* = 0.014]	Ticagrelor monotherapy between 12 and 24 m following initial therapy, compared to aspirin, significantly reduced the primary endpoint and the risk of MI

Data are presented in the format (intervention/control group). ST, stent thrombosis; TVR, target vessel revascularization; m, months; CV, cardiovascular; MI, myocardial infarction; DES, drug eluting stent; BMS, bare metal stent; CI, confidence interval; DAPT, dual antiplatelet therapy; TIMI, thrombolysis in myocardial infarction; BARC, Bleeding Academic Research Consortium.

**Table 5.** **Risk of myocardial infarction and stent thrombosis**.

Trial	MI [hazard ratio (95% CI)]	*p*	ST (definite or probable). [hazard ratio (95% CI)]	*p*
Studies on extended DAPT				
ARCTIC – Interruption [10]	9 (1%)/9 (1%) [1.04 (0.41–2.62)]	*p* = 0.94	0/3 (1%)	-
ITALIC [11]	9 (1%)/12 (1.3%) [1.335 (0.562–3.167)]	*p* = 0.513	3 (0.3%)/6 (0.6%) [1.995 (0.499–7.976)]	*p* = 0.329
OPTIDUAL [12]	11 (1.6%)/16 (2.3%) [0.67 (0.31–1.44)]	*p* = 0.31	3 (0.4%)/1 (0.1%) [2.97 (0.31–28.53)]	*p* = 0.35
DAPT (BMS) [13]	22 (2.7%)/25 (3.1%) [0.91 (0.51–1.62)]	*p* = 0.74	definite 4 (0.5%)/9 (1.11%) [0.49 (0.15–1.64)]	*p* = 0.24
DAPT (DES) [14]	99 (2.1%)/198 (4.1%) [0.47 (0.37–0.61)]	*p * < 0.001	19 (0.4%)/65 (1.4%) [0.29 (0.17–0.48)]	*p * < 0.001
DES LATE [15]	19 (0.8%)/27 (1.2%) [1.43 (0.80–2.58)]	*p* = 0.23	definite 7 (0.3%)/11 (0.5%) [1.59 (0.61–4.09)]	*p* = 0.34
NIPPON [16]	Non fatal 1 (0.1%)/4 (0.2%) [–0.2 (–0.6–0.1)]	*p* = 0.37	1 (0.1%)/2 (0.1%) [–0.1 (–0.4–0.2)]	*p* = 1.00
REAL-LATE and ZEST-LATE [17]	10 (0.8%)/7 (0.7%) [1.41 (0.54–3.71)]	*p* = 0.49	definite 5 (0.4%)/4 (0.4%) [1.23 (0.33–4.58)]	*p* = 0.76
PRODIGY [18]	39 (4.0%)/41 (4.2%) [1.06 (0.69–1.63)]	*p* = 0.80	definite 8 (0.8%)/7 (0.7%) [0.88 (0.32–2.42)]	*p* = 0.80
PEGASUS-TIMI 54 [19]	ticagrelor 90 mg: (4.33%)/(5.18%) [0.79 (0.66–0.95)]	90 mg: *p* = 0.012	definite ticagrelor 90 mg: (0.5%)/(0.71%) [0.6 (0.35–1.01)] ticagrelor 60 mg: (0.64%)/(0.71%) [0.94 (0.59–1.49)]	90 mg: *p* = 0.055
	ticagrelor 60 mg: (4.47%)/(5.18%) [0.84 (0.7–1.0)]	60 mg: *p* = 0.046		60 mg: *p* = 0.793
THEMIS-PCI [20]	155 (3%)/208 (4%) [0.76 (0.61–0.93)]	*p* = 0.008	NS	-
Studies on long-term P2Y12 inhibitor monotherapy				
HOST-EXAM Extended [21]	Non fatal, 40 (1.6%)/53 (2.3%) [0.71 (0.47–1.07)]	*p* = 0.102	12 (0.5%)/17 (0.7%) [0.67 (0.32–1.39)]	*p* = 0.28
SMART-CHOICE 3 [22]	23 (1%)/42 (2.2%) [0.54 (0.33–0.9)]	*p * < 0.05	definite or probable 1 (0%)/5 (0.2%) [0.20 (0.02–1.68)]	*p * > 0.05
GLOBAL LEADERS [23]	37 (0.7%)/71 (1.2%) [0.57 (0.38–0.85)]	*p* = 0.006	definite or probable 10 (0.2%)/16 (0.3%) [0.68 (0.31–1.51)]	*p* = 0.347

Data are presented as (intervention/control group). In SMART-CHOICE 3 trial the *p* values for myocardial infarction and stent thrombosis are not stated in text, but they are mentioned as significant or non-significant. MI, myocardial infarction; ST, stent thrombosis; DES, drug eluting stent; BMS, bare metal stent; CI, confidence interval; NS, not stated; DAPT, dual antiplatelet therapy; TIMI, thrombolysis in myocardial infarction.

**Table 6.** **Bleeding classification and incidence**.

Trial	Bleeding classification	Bleeding incidence	Hazard ratio (95% CI), *p*
Studies on extended DAPT			
ARCTIC-Interruption [10]	STEEPLE major	1%/(<0.5%)	0.15 (0.02–1.20), *p* = 0.07
ITALIC [11]	TIMI major	4 (0.4%)/0	Not applicable
OPTIDUAL [12]	TIMI major	4 (0.6%)/4 (0.6%)	*p* = 1.00
DAPT (BMS) [13]	BARC	Type 2, 3 or 5: 36 (4.56%)/14 (1.8%)	Type 2, 3 or 5: *p* = 0.002
		Type 5: 0/1	Type 5: *p* = 0.31
DAPT (DES) [14]	BARC	Type 2, 3 or 5: 263 (5.6%)/137 (2.9%)	Type 2, 3 or 5: *p * < 0.001
		Type 5: 7/4	Type 5: *p* = 0.38
DES LATE [15]	TIMI major	34 (1.4%)/24 (1.1%)	0.71 (0.42–1.2), *p* = 0.2
NIPPON [16]	BARC	Type 3 or 5: 12 (0.7%)/11 (0.7%)	Type 3 or 5: 0.1 (–0.6–0.7), *p* = 0.83
		Type 5: 2/0	
REAL-LATE and ZEST-LATE [17]	TIMI major	3 (0.2%)/1 (0.1%)	2.96 (0.31–28.46), *p* = 0.35
PRODIGY [18]	TIMI major	16 (1.6%)/6 (0.6%)	0.38 (0.15–0.97), *p* = 0.041
PEGASUS-TIMI 54 [19]	TIMI major	ticagrelor 90 mg (2.7%)/(1.05%)	ticagrelor 90 mg : 2.86 (2.01–4.08), *p * < 0.001
		ticagrelor 60 mg (2.46%)/(1.05%)	ticagrelor 60 mg : 2.45 (1.71–3.5), *p * < 0.001
THEMIS-PCI [20]	TIMI major	122/84	1.51 (1.14–1.99), *p * < 0.05
Studies on long-term P2Y12 inhibitor monotherapy			
HOST-EXAM Extended [21]	BARC type 2, 3 or 5	62 (2.6%)/90 (3.9%)	0.65 (0.47–0.90), *p* = 0.008
SMART-CHOICE 3 [22]	BARC	Type 2, 3 or 5: 53 (3%)/55 (3%)	Type 2, 3 or 5: 0.97 (0.67–1.42)
		Type 3 or 5: 26 (1.6%)/26 (1.3%)	Type 3 or 5: 1.00 (0.58–1.73)
GLOBAL LEADERS [23]	BARC	Type 2, 3 or 5: 94 (1.8%)/68 (1.2%)	Type 2, 3 or 5: 1.52 (1.11–2.08), *p* = 0.009
		Type 3 or 5: 28 (0.5%)/17 (0.3%)	Type 3 or 5: 1.80 (0.99–3.30), *p* = 0.055

Data are presented in the format (intervention/control group). In SMART-CHOICE 3 trial the *p* values for bleeding outcomes are intentionally not stated by the authors to avoid misinterpretation of statistical significance, as bleeding outcomes were secondary endpoints; DES, drug eluting stent; BMS, bare metal stent; CI, confidence interval; DAPT, dual antiplatelet therapy; STEEPLE, safety and efficacy of enoxaparin in PCI patients, an international randomized evaluation; TIMI, thrombolysis in myocardial infarction; BARC, Bleeding Academic Research Consortium.

### 3.3 Heterogeneity of Included Studies

The included studies exhibited significant heterogeneity across clinical, procedural, and methodological domains. The systematic review included eleven RCTs, alongside two prespecified analyses and one post hoc analysis of RCTs [10, 11, 12, 13, 14, 15, 16, 17, 18, 19, 20, 21, 22, 23]. Trial designs differed in blinding, with some conducted as double-blind and others open-label (Table 2), potentially influencing risk of bias. Baseline demographics were generally comparable across studies, with similar age distributions, diabetes prevalence, and smoking history, although prior MI rates ranged notably, from 0% in THEMIS-PCI selected diabetic cohort up to 46% in SMART-CHOICE 3. Included trials covered diverse geographic regions and demonstrated wide variation in follow-up durations, ranging from 18 months to nearly 6 years. Considerable heterogeneity was also evident regarding the duration, type, and sequencing of both initial DAPT and long-term antiplatelet regimens compared to aspirin monotherapy (Table 2). Procedural characteristics varied broadly with PCI indications ranging from elective interventions (e.g., ARCTIC-Interruption) to ACS presentations and stent types ranging from bare-metal stents to several types of drug-eluting stents (Table 3). Primary endpoints were generally consistent and comprised composite ischemic outcomes with all-cause mortality, MI, and stroke; however, several studies incorporated additional components such as urgent revascularization or major bleeding, adding variability that complicates cross-trial comparisons (Table 4). Importantly, ischemic outcomes like MI and definite or probable stent thrombosis were uniformly reported and presented separately to facilitate meaningful comparisons (Table 5). Moreover, bleeding outcomes differed in classification methods, as detailed in Table 6, which limits direct comparability of safety data. More detailed information on study design, clinical and procedural characteristics, outcomes, and bleeding classification systems utilized in each study can be found in Tables 1,2,3,4,5,6.

Patient populations also showed substantial variation in risk profiles. Several trials focused specifically on populations with high thrombotic risk. PEGASUS-TIMI 54, THEMIS-PCI, and SMART-CHOICE 3 each selected patients meeting high-risk criteria based on clinical and procedural characteristics detailed in subsection 3.2 [19, 20, 22]. Conversely, other large RCTs, including ARCTIC-Interruption, ITALIC, DAPT, DES LATE, PRODIGY, NIPPON and HOST-EXAM, enrolled all-comer CCS patients post-PCI without selective restriction to high-risk subgroups and these trials performed subgroup analyses based on clinical features such as age, diabetes, or PCI complexity [10, 11, 13, 14, 15, 16, 18, 21]. Moreover, two RCTs did not conduct subgroup analyses and reported only aggregate outcomes for their overall populations [12, 17]. This variability in patient selection and subgroup focus contributes to important clinical heterogeneity influencing both ischemic and bleeding outcomes.

Together, these clinical, procedural, and methodological differences underscore the complexity of balancing ischemic benefits against bleeding risks, highlighting the necessity of individualized patient risk stratification and tailored therapeutic strategies in interpreting and applying the evidence.

### 3.4 Insights From Observational Data and Risk Stratification

Although observational studies were excluded from this systematic review, numerous such studies have explored whether specific clinical or angiographic characteristics may identify patients with a history of PCI who would benefit from prolonged DAPT. For example, three observational studies demonstrated that DAPT with clopidogrel beyond 12 months provided benefit in patients undergoing PCI of the left main coronary artery [33, 34, 35]. Similarly, four studies reported benefit in ischemic endpoints with prolonged DAPT in patients undergoing left main bifurcation or complex PCI [36, 37, 38, 39]. In contrast, one trial found no benefit in patients undergoing treatment for chronic total occlusion of coronary arteries [40]. Other studies evaluated the potential benefit based on patient comorbidities. Prolonged DAPT was associated with better outcomes in diabetic patients, but not in those with anemia or chronic kidney disease on dialysis [41, 42, 43, 44]. Moreover, no benefit was observed in patients presenting with ACS, whereas one study noted significant benefit in patients with elevated lipoprotein(a) undergoing PCI [45, 46, 47].

These observational studies, despite their limitations, reflect a growing effort to refine the optimal, individualized antiplatelet strategy. There is ongoing interest to identify specific patient subpopulations defined by biomarkers or clinical and angiographic characteristics, who may benefit from intensified antiplatelet treatment. This direction is consistent with the findings of this systematic review and a recent meta-analysis by Elliott *et al*. [7], which reported that patients with prior MI, those younger than 75 years, and individuals presenting with ACS may benefit from prolonged DAPT. Still, they highlight the importance of careful patient selection when considering long-term intensive therapy.

Personalization of antiplatelet treatment is crucial, as unjustified prolongation of DAPT increases bleeding risk, while premature discontinuation of DAPT may lead to adverse cardiovascular events [48]. To support individualization, various risk scores have been developed using patient data to predict who may benefit from DAPT extension [5]. Some of the most commonly used are the PRECISE-DAPT DAPT, and PARIS risk scores, which help guide the decision to continue therapy based on clinical parameters [9, 49]. Even though these tools were initially validated in Western populations, their predictive accuracy has been questioned in specific populations including those of Sweden and China [50, 51]. In contrast, recent evidence highlights the central role of bleeding risk scores, particularly PRECISE-DAPT and ARC-HBR, in guiding antiplatelet therapy selection and duration after PCI. The PRECISE-DAPT score, which incorporates clinical and laboratory parameters, identifies patients at high bleeding risk (score ≥25) who may benefit from abbreviated DAPT durations without increasing ischemic events. Similarly, the ARC-HBR criteria provide standardized definitions for high bleeding risk, including factors such as advanced age, anemia, prior major bleeding, and chronic kidney disease. Applying these tools enables a more individualized approach to balancing ischemic and bleeding risks and supports tailoring antiplatelet strategies to the patient’s risk profile. In clinical practice, this often translates to shortening DAPT duration to 1–3 months in high bleeding risk patients. Such abbreviated DAPT regimens may be followed by single antiplatelet therapy, often with a P2Y12 inhibitor rather than aspirin, or even by initiation of aspirin-free strategies consisting of potent P2Y12 inhibitors immediately post-PCI, as supported by recent trials. These evolving strategies emphasize precision medicine, ensuring therapy intensity and duration align with individual patient risk profiles. As patients meeting high bleeding risk criteria constitute a substantial subset in contemporary practice, the use of validated risk scores combined with these tailored therapeutic options is now considered essential for optimal, patient-centered decision-making [52].

While optimal antiplatelet therapy remains central to reducing ischemic risk post-PCI, it is critical to recognize that comprehensive management of all modifiable thrombotic risk factors is essential for improving cardiovascular outcomes in all patients and particularly so in those at high bleeding risk, who may not tolerate intensified antiplatelet regimens. Among these factors, individualized lipid management plays a pivotal role. High-intensity statins are first-line in order to effectively lower low-density lipoprotein cholesterol (LDL-C) levels and reduce atherosclerotic cardiovascular risk, often combined early with ezetimibe or proprotein convertase subtilisin/kexin type 9 (PCSK9) inhibitors as needed to achieve LDL-C targets. Lipid-lowering therapies should be personalized based on patient comorbidities, prior treatment tolerance, and risk profile. In patients with statin intolerance or inadequate responses or genetic lipid disorders, newer agents such as bempedoic acid or emerging RNA-based therapies targeting lipoprotein(a), apolipoprotein C3, and ANGPTL3 offer additional promising options. By addressing residual lipid-mediated risk factors, clinicians can optimize secondary prevention beyond antiplatelet therapy alone. Incorporating individualized lipid control into the broader risk factor modification plan embodies a patient-centered approach essential for long-term management in CCS populations [53].

At present, the decision to pursue alternative long-term therapy remains an area of active research [54]. While the role of aspirin in secondary prevention is well established, robust results from large, randomized, international, double-blind clinical trials are necessary in order to determine the optimal long-term antiplatelet therapy to individual patient profiles.

### 3.5 Limitations

This systematic review has several limitations, primarily related to the heterogeneity and methodological differences among the included trials. There was considerable variability in patient comorbidities, PCI indications, comparator antiplatelet regimens, treatment duration and definitions of both primary efficacy and bleeding outcomes. Except for the PRODIGY trial, most studies enrolled only patients who remained free of ischemic and bleeding events during the standard DAPT period. As a result, higher-risk patients were excluded, thus limiting generalizability. Additionally, the availability of data for subgroup analyses from the trials was limited, restricting the ability to draw robust conclusions regarding specific patient subgroups.

## 4. Conclusion

Long-term intensified antiplatelet therapy may provide benefit in patients with CCS and a history of PCI. Specific subgroups with high thrombotic burden, such as those with a history of acute myocardial infarction, diabetes mellitus, peripheral artery disease or complex coronary artery disease may derive even greater benefit, provided they are not at increased risk for bleeding. These findings highlight the importance of carefully balancing ischemic protection against bleeding risk, to achieve optimal, individualized therapy.

## Availability of Data and Materials

All data relevant to the study are included in the article or uploaded as supplementary information.

## Supplementary Material

Supplementary material associated with this article can be found, in the online version, at https://doi.org/10.31083/RCM44227.
