# Supplementary material for: Antiplatelet Therapy in Chronic Coronary Artery Disease Patients With a History of Angioplasty. When is Aspirin Not Enough? A Systematic Review
Source: Rev Cardiovasc Med. 2025 Sep 24;26(9):44227. doi: 10.31083/RCM44227 (PMC12516749; doi:10.31083/RCM44227)
Supplement: Supplementary file 1 [file 2153-8174-26-9-44227-s1.zip › PRISMA_2020_checklist.docx]

| **Section and Topic** | **Item #** | **Checklist item** | **Location where item is reported** |
| --- | --- | --- | --- |
| **TITLE** | | |  |
| Title | 1 | Identify the report as a systematic review. | Page 1. The title identifies the report as a systematic review |
| **ABSTRACT** | | |  |
| Abstract | 2 | See the PRISMA 2020 for Abstracts checklist. | Page 1. The abstract includes a background, methods, results, conclusion and PROSPERO registration section and has been prepared in accordance with the PRISMA checklist |
| **INTRODUCTION** | | |  |
| Rationale | 3 | Describe the rationale for the review in the context of existing knowledge. | Page 2 |
| Objectives | 4 | Provide an explicit statement of the objective(s) or question(s) the review addresses. | Page 2 |
| **METHODS** | | |  |
| Eligibility criteria | 5 | Specify the inclusion and exclusion criteria for the review and how studies were grouped for the syntheses. | Page 2. 2.2 + 2.4 paragraphs |
| Information sources | 6 | Specify all databases, registers, websites, organisations, reference lists and other sources searched or consulted to identify studies. Specify the date when each source was last searched or consulted. | Page 2. 2.3. PubMed, the Cochrane Library, ClinicalTrials.gov, reference lists of relevant European and American guidelines. Last search on 29 May 2025. |
| Search strategy | 7 | Present the full search strategies for all databases, registers and websites, including any filters and limits used. | Page 2. 2.3 + Full search available on reasonable request |
| Selection process | 8 | Specify the methods used to decide whether a study met the inclusion criteria of the review, including how many reviewers screened each record and each report retrieved, whether they worked independently, and if applicable, details of automation tools used in the process. | Page 2. 2.3. Two reviewers independently screened the literature. Discrepancies were resolved between them and if necessary, by a third reviewer |
| Data collection process | 9 | Specify the methods used to collect data from reports, including how many reviewers collected data from each report, whether they worked independently, any processes for obtaining or confirming data from study investigators, and if applicable, details of automation tools used in the process. | Page 2. 2.4. Data were independently extracted by two reviewers using a predefined Google Sheet |
| Data items | 10a | List and define all outcomes for which data were sought. Specify whether all results that were compatible with each outcome domain in each study were sought (e.g. for all measures, time points, analyses), and if not, the methods used to decide which results to collect. | Page 2. 2.4. We extracted data for the following : all-cause mortality, cardiovascular mortality, MI, stent thrombosis and bleeding events. For each outcomes, we extracted the results reported at the longest follow up available in each study and reported what that time point was. We included all available subgroup analyses reported for each outcome. We reported any measurement tool that was reported by each study authors. |
|  | 10b | List and define all other variables for which data were sought (e.g. participant and intervention characteristics, funding sources). Describe any assumptions made about any missing or unclear information. | Page 2. 2.4. We extracted the following data : study design, country, sample size, age, sex, comorbidities, intervention details, comparator details, follow up period. If data were missing or were unclear, we searched each study’s supplementary materials. No assumptions were made about missing values. If data were missing or were unclear, we noted that information. We did not contact study authors for clarification |
| Study risk of bias assessment | 11 | Specify the methods used to assess risk of bias in the included studies, including details of the tool(s) used, how many reviewers assessed each study and whether they worked independently, and if applicable, details of automation tools used in the process. | Pages 2-3. 2.5. The RoB 2.0 tool was used to assess the risk of bias in the included studies |
| Effect measures | 12 | Specify for each outcome the effect measure(s) (e.g. risk ratio, mean difference) used in the synthesis or presentation of results. | Pages 2 and 6-12. 2.4 & Tables 4, 5, 6. For all outcomes, including mortality, myocardial infarction, stent thrombosis, bleeding, we extracted Hazard Ratios (HRs), 95% confidence intervals and p-values. We did not perform a meta-analysis, therefore the results were presented narratively. |
| Synthesis methods | 13a | Describe the processes used to decide which studies were eligible for each synthesis (e.g. tabulating the study intervention characteristics and comparing against the planned groups for each synthesis (item #5)). | Pages 7-12 (Tables of Results section) Detailed key characteristics of the antiplatelet regimen used in each study are presented in Table 4. No specific grouping was required, as the intervention characteristics made it possible to distinguish the relevant comparisons for each outcome. |
|  | 13b | Describe any methods required to prepare the data for presentation or synthesis, such as handling of missing summary statistics, or data conversions. | Pages 7-12. (Tables of Results section) No conversions were performed. All data were extracted as reported in the original studies. |
|  | 13c | Describe any methods used to tabulate or visually display results of individual studies and syntheses. | Pages 7-12. (Tables of Results section) Characteristics of each included study and outcomes are presented in the relevant Table. There are also figures presenting the risk of bias assessment and PRISMA flow diagram |
|  | 13d | Describe any methods used to synthesize results and provide a rationale for the choice(s). If meta-analysis was performed, describe the model(s), method(s) to identify the presence and extent of statistical heterogeneity, and software package(s) used. | Meta-analysis was not performed due to heterogeneity among included studies |
|  | 13e | Describe any methods used to explore possible causes of heterogeneity among study results (e.g. subgroup analysis, meta-regression). | No formal statistical methods were used to explore heterogeneity. Potential sources of variations between studies, such as differences in antiplatelet regimens, study populations, follow-up and outcome definitions or bleeding classification, were explored narratively and describes in the systematic review. |
|  | 13f | Describe any sensitivity analyses conducted to assess robustness of the synthesized results. | Sensitivity analysis was not performed. |
| Reporting bias assessment | 14 | Describe any methods used to assess risk of bias due to missing results in a synthesis (arising from reporting biases). | Page 2. Evaluated by comparing original study protocols to the reported outcomes |
| Certainty assessment | 15 | Describe any methods used to assess certainty (or confidence) in the body of evidence for an outcome. | Page 3. Most of the included studies were judged as ‘low risk’ of bias. One trial was judged as ‘high risk’ of bias due to its post-hoc nature. |
| **RESULTS** | | |  |
| Study selection | 16a | Describe the results of the search and selection process, from the number of records identified in the search to the number of studies included in the review, ideally using a flow diagram. | PRISMA flow diagram (Figure 1) and first paragraph of results section (Page 7) |
|  | 16b | Cite studies that might appear to meet the inclusion criteria, but which were excluded, and explain why they were excluded. | Page 2. 2.2 Paragraph and Observational Studies paragraph in combined Results and Discussion section (Page 6) |
| Study characteristics | 17 | Cite each included study and present its characteristics. | Page 4, Table 1 |
| Risk of bias in studies | 18 | Present assessments of risk of bias for each included study. | Risk of bias was assessed using RoB 2 tool, available in Supplementary material |
| Results of individual studies | 19 | For all outcomes, present, for each study: (a) summary statistics for each group (where appropriate) and (b) an effect estimate and its precision (e.g. confidence/credible interval), ideally using structured tables or plots. | Pages 6-10. Tables 4,5,6. |
| Results of syntheses | 20a | For each synthesis, briefly summarise the characteristics and risk of bias among contributing studies. | Pages 4-5. Study characteristics and designs are presented in Tables 1 and 2. Risk of bias was assessed for all included studies and one study was judged to be “high risk” due to its post hoc design (Page 3) |
|  | 20b | Present results of all statistical syntheses conducted. If meta-analysis was done, present for each the summary estimate and its precision (e.g. confidence/credible interval) and measures of statistical heterogeneity. If comparing groups, describe the direction of the effect. | No statistical synthesis or meta-analysis was performed |
|  | 20c | Present results of all investigations of possible causes of heterogeneity among study results. | No formal investigations of heterogeneity were conducted, as a meta-analysis was not performed. Differences between studies were instead explored narratively. |
|  | 20d | Present results of all sensitivity analyses conducted to assess the robustness of the synthesized results. | No sensitivity analyses were conducted, as a meta-analysis was not performed. Results were synthesized narratively, and the robustness of the findings was discussed in relation to study quality and consistency across the included studies. |
| Reporting biases | 21 | Present assessments of risk of bias due to missing results (arising from reporting biases) for each synthesis assessed. | Formal assessments of risk of bias due to missing results were not conducted. Potential reporting biases were considered narratively based on study characteristics. |
| Certainty of evidence | 22 | Present assessments of certainty (or confidence) in the body of evidence for each outcome assessed. | Pages 2-3. The overall confidence in the findings is moderate-high. Most included studies were judged to be at low risk of bias except one study which was assessed as having high risk of bias, but this study did not alter the conclusions. Differences in follow-up durations may affect outcome comparability. Formal assessments of publication bias were not conducted due to lack of meta-analysis. |
| **DISCUSSION** | | |  |
| Discussion | 23a | Provide a general interpretation of the results in the context of other evidence. | Pages 10-13. The findings of this systematic review suggest that extended, intensified antiplatelet treatment may provide benefit in patients with high thrombotic burden. These results align with prior Guidelines of ESC. While most included studies were at low risk of bias, variations in patient populations, antiplatelet regimens and follow-up periods introduce some uncertainty. Nonetheless, the consistency of findings across diverse settings strengthens confidence in the overall conclusions. Further research is needed. |
|  | 23b | Discuss any limitations of the evidence included in the review. | Pages 10-13. Heterogeneity among studies, risk of bias in the included studies, lack of meta-analysis, potential publication bias. |
|  | 23c | Discuss any limitations of the review processes used. | No meta-analysis was conducted, data extraction relied on reported results and no attempts were made to contact authors for missing information. Narrative synthesis may be more prone to interpretation bias compared to quantitative methods. |
|  | 23d | Discuss implications of the results for practice, policy, and future research. | Pages 12-13. The findings provide valuable insights into the comparative effectiveness and safety of intensified antiplatelet regimens, supporting clinicians in making informed decisions tailored to patients’ characteristics. Individualization of therapy remains important. High quality trials should be designed, and further research is warranted to address the existing heterogeneity and limitations. There is also need for longer follow-up periods to assess long-term benefits and risks |
| **OTHER INFORMATION** | | |  |
| Registration and protocol | 24a | Provide registration information for the review, including register name and registration number, or state that the review was not registered. | Page 1. CRD420251069004, <https://www.crd.york.ac.uk/PROSPERO/view/CRD420251069004> |
|  | 24b | Indicate where the review protocol can be accessed, or state that a protocol was not prepared. | Page 1. The protocol for this systematic review is registered and can be accessed on the PROSPERO database. |
|  | 24c | Describe and explain any amendments to information provided at registration or in the protocol. | The review was conducted as originally planned. |
| Support | 25 | Describe sources of financial or non-financial support for the review, and the role of the funders or sponsors in the review. | Does not apply |
| Competing interests | 26 | Declare any competing interests of review authors. | No competing interests |
| Availability of data, code and other materials | 27 | Report which of the following are publicly available and where they can be found: template data collection forms; data extracted from included studies; data used for all analyses; analytic code; any other materials used in the review. | The protocol can be accessed on the PROSPERO database. Detailed methods for the literature search and risk of bias assessment are available upon reasonable request. |

*From:*  Page MJ, McKenzie JE, Bossuyt PM, Boutron I, Hoffmann TC, Mulrow CD, et al. The PRISMA 2020 statement: an updated guideline for reporting systematic reviews. BMJ 2021;372:n71. doi: 10.1136/bmj.n71. This work is licensed under CC BY 4.0. To view a copy of this license, visit <https://creativecommons.org/licenses/by/4.0/>
