# Supplementary material for: Antiplatelet Therapy in Chronic Coronary Artery Disease Patients With a History of Angioplasty. When is Aspirin Not Enough? A Systematic Review
Source: Rev Cardiovasc Med. 2025 Sep 24;26(9):44227. doi: 10.31083/RCM44227 (PMC12516749; doi:10.31083/RCM44227)
Supplement: Supplementary file 1 [file 2153-8174-26-9-44227-s1.zip › Supplementary Fig. 1.pdf]

Study	Randomization Process	Deviations from Interventions	Missing Outcome Data	Measurement of Outcome	Selection of Reported Result	Overall RoB
ARCTIC - Interruption	Low	Low	Low	Low	Low	Low
ITALIC	Low	Low	Low	Low	Low	Some concerns[1]
OPTIDUAL	Low	Low	Low	Low	Low	Low
HOST-EXAM Extended	Low	Low	Low	Low	Low	Low
DAPT BMS	Low	Low	Low	Low	Some concerns	Some concerns
DAPT DES	Low	Low	Low	Low	Low	Low
DES LATE	Low	Low	Low	Low	Low	Low
NIPPON	Low	Some concerns	Low	Low	Low	Some concerns
REAL LATE & ZEST-LATE	Low	Low	Low	Low	Low	Low
PRODIGY	Low	Low	Low	Low	Low	Low
PEGASUS-TIMI 54	Low	Low	Low	Low	Low	Low
THEMIS-PCI	Low	Low	Low	Low	Low	Low
GLOBAL LEADERS	High	Some concerns	Some concerns	Some concerns	Some concerns	High
SMART-CHOICE 3	Low	Low	Low	Low	Low	Low
						Open-label trials were judged to be at low risk of bias despite a lack of blinding if the outcomes were objectively assessed.
